# Olive Pâté by Multi-Phase Decanter as Potential Source of Bioactive Compounds of Both Nutraceutical and Anticancer Effects

**DOI:** 10.3390/molecules25245967

**Published:** 2020-12-16

**Authors:** Barbara Lanza, Martina Cellini, Sara Di Marco, Emira D’Amico, Nicola Simone, Lucia Giansante, Arianna Pompilio, Giuseppina Di Loreto, Martina Bacceli, Paolo Del Re, Giovanni Di Bonaventura, Luciana Di Giacinto, Gitana Maria Aceto

**Affiliations:** 1 Council for Agricultural Research and Economics (CREA), Research Centre for Engineering and Agro-Food Processing (CREA-IT), Via Lombardia, 65012 Cepagatti, Italy; martina.cellini1992@gmail.com (M.C.); sarettadimarco87@gmail.com (S.D.M.); nicola.simone@crea.gov.it (N.S.); lucia.giansante@crea.gov.it (L.G.); giuseppina.diloreto@crea.gov.it (G.D.L.); martina.bacceli@crea.gov.it (M.B.); paolo.delre@crea.gov.it (P.D.R.); luciana.digiacinto@crea.gov.it (L.D.G.); 2 Department of Medical, Oral and Biotechnological Sciences, “G. d’Annunzio” University of Chieti-Pescara, Via dei Vestini 31, 66100 Chieti, Italy; emira.damico@unich.it (E.D.); arianna.pompilio@unich.it (A.P.); giovanni.dibonaventura@unich.it (G.D.B.); gitana.aceto@unich.it (G.M.A.); 3 Center for Advanced Studies and Technology (CAST), “G. d’Annunzio” University of Chieti-Pescara, Via Luigi Polacchi 11, 66100 Chieti, Italy

**Keywords:** olive oil by-products, phenolic compounds, multi-phase decanter, colon cancer cells, secoiridoids

## Abstract

In the oil sector, a novelty in the centrifugal extraction system is represented by the multi-phase decanters (DMF) that work without adding process water and with the advantage of recovering a dried pomace and a by-product, called “pâté”, consisting of the pulp and its vegetation water, without traces of stone. The pâté has a high content of phenolic compounds, mainly represented by secoiridoids and verbascoside. The present work investigated the efficacy of two different ways of debittering (by sequential filtrations and spontaneous fermentation) of DMF pâté from three olive cultivars (*Olea europaea* L. “Leccino”, “Carboncella” and “Tortiglione”) to make the pâté edible, and, contemporary, investigated also the effect of its phenolic bioactive extracts on pathogenic bacteria and colon cancer cell model. Daily filtrations of pâté of the three cultivars have been shown to be more efficient in phenolic degradation. The activity of the indigenous microflora on the other hand takes a longer time to degrade the phenolic component and therefore to de-bitter it. None of pâté showed antibacterial activity. Colorimetric assay MTS for cell viability and metabolic activity tested on colon cancer cells Caco-2 and HCT116 suggest a potential beneficial effect of the dried extracts probably related to the modulation of gene expression under these treatments.

## 1. Introduction

**Figure 1:**
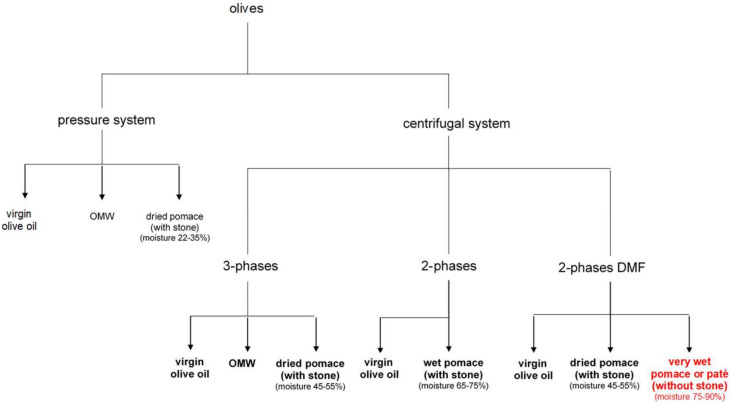
Scheme of olive pomace production. OMW = olive mill wastewater.

Pomace is the solid by-product obtained from the mechanical extraction of virgin olive oil (Figure 1). Currently, in Italy, the pomace obtained by the pressure system is used almost exclusively for the residual oil recovery, while there are no great requests from the pomace mills of the pomace obtained by the continuous system for centrifugation and, specially, for the one deriving from the 2-phase decanter [1]. The composition of the pomace is variable and mainly depends on the extraction system adopted, along with the variety and the degree of ripeness of the fruit. Currently it represents more a problem than a resource for the mill because of the presence of large quantities of polyphenols and stone (or kernel) and, therefore, lignin. In order to obtain a pomace without stone, there are basically three alternatives to the classic processes: (a) separation of the stone upstream of the plant; (b) separation of the stone downstream of the plant, and (c) separation of the stone with multi-phase decanter.

The stoner to be used upstream of the plant has the function of continuously pitting the olives obtaining the whole core as a by-product and the pulp alone from which to extract the oil (pitted oil and pomace without kernel). The pitting technique produces oils with a lower number of peroxides and greater forced resistance to oxidation, compared to the oils obtained from whole-grain pasta, the absence of fragments and almond leads to a reduction of lipolytic and oxidative phenomena with a consistent improvement on the shelf life of the finished product.

Some mills have tried to separate downstream the wet pomace into its two fractions (kernel and fiber) with a stone-separator machine, in order to use the kernel as fuel. In particular, the highest income is obtained when the olives are processed with a 2-phase decanter because a very moist pomace is produced (humidity > 60%) which favors a higher efficiency of the separating machine and a consequent higher recovery of kernel. The machines on the market could process around 80–200 quintals/h in two or three phases. The substantial difference between these two methods could be due to the humidity of the pomace (with kernel): in the case of two-phase processing, in fact, the humidity can vary from 65% to 75%, in the case of three-phase processing, instead, the pomace is drier with an estimated humidity of around 45–55% (dried pomace).

A novelty in the two-phase extraction system is represented by the multi-phase decanters (DMF) that work without adding process water and with the advantage of recovering a dried pomace similar to that coming from the three phases (45–55% of humidity) to be used as fuel for stoves and boilers and a by-product, called “pâté“, consisting of the pulp and its vegetation water, without traces of kernel [2]. The pâté has a high content of organic substance, a good fiber and raw protein content and a low lignin content, comparable to the pomace obtained from pitted olives. It also has a high content of phenolic compounds mainly represented by secoiridoids at low, medium and high molecular weight [2,3,4], and a fat residue (3–6%) whose acidic composition is like that of an extra virgin olive oil [4,5]. In addition, it has high humidity, on average between 75 and 90%. It therefore proves to be a product with high added value for the oil mill, being able to be reused in a competitive way in different fields: agronomic (as a soil fertilizer), zootechnical (as a supplement in the feeding of cattle and sheep, enriches milk and derivatives of polyunsaturated fatty acids and antioxidant polyphenols), energy (as biomass for biogas plants). Its main properties have been reported in many studies that have highlighted its biological activities. Specifically, their effects on human health are anti-inflammatory, anti-hyperglycemic, hepatoprotective, cardioprotective, antimicrobial and anticancer [6,7,8]. Recent studies [9] suggest that the main biophenols found in *Olea europaea* L. products and sub-products may be used as a potential source of both antioxidant and anticancer effects, and in particular, in ovarian cancer that is characterized by a high mortality rate among gynecological malignancies worldwide.

This new olive mill by product is also potentially suitable for human consumption but, due to its phenolic content which gives it a bitter taste, without any treatment it is not edible, so this olive paste needs transformation [3].

The aim of this work was to investigate the profile modification of the biophenolic pattern following sequential filtrations and spontaneous fermentation to make the pâté edible and, contemporary, to investigate also the effect of pâté phenolic bioactive extracts on colon cancer cell model and pathogenic bacteria.

## 2. Results

### 2.1. Chemical Characterization of Fresh Olive Pâté

Table 1 shows the chemical characteristics of fresh pate. Leccino pâté shows the lowest titratable acidity and the highest pH. Tortiglione pâté shows the highest umidity (> 86%) and the lowest oil content. Carboncella shows the highest content in ash. Carboncella and Tortiglione are very rich in total biophenols (5899 and 5543 mg/kg of oil, respectively) with respect to Leccino cv. (948 mg/kg).

In Table 2 is reported the amount of individual phenolic compounds identified in Leccino, Carboncella and Tortiglione pâté. Oleacein, hydroxytyrosol and verbascoside are the most abundant phenols.

### 2.2. Biophenolic Degradation by Test A and Test B

Test A aims to debitter DMF pâté through daily filtrations: the test started with the filtration of the fresh pâté obtained from the DMF decanter; then, the filtered product was mixed with an equal amount of water and, after 24 h, was filtered again. The same procedure was repeated for five times (see Section 4.3). Test B, on the other hand, did not undergo any daily external stimulus, but the pâté was filtered once every seven days to monitor the degree of spontaneous debittering achieved (see Section 4.4). In Table 3 is showed the comparison of degradation rate of total biophenols during Test A and Test B. In relation to the degradation of phenolic compounds, Test A is much faster than Test B. In Test A, Leccino cultivar shows the slowest phenolic degradation (after 24 h 86% of the total phenols remain; after 48 h the 70%; after 72 h 58% and does not reach zero). The phenolic degradation in Carboncella is the fastest of the three samples considered: after 24 h only 43% remains and after 72 h only 10% remains. Finally, Tortiglione reaches values close to zero after 72 h. Test B shows the same trend but with a lower degradation rate. In Leccino the phenolic compounds do not degrade more than 45% at the end of Test B. Carboncella shows a residual equal to 10% after just 14 days, and Tortiglione reaches the same value as the latter after 21 days.

**Figure 2:**
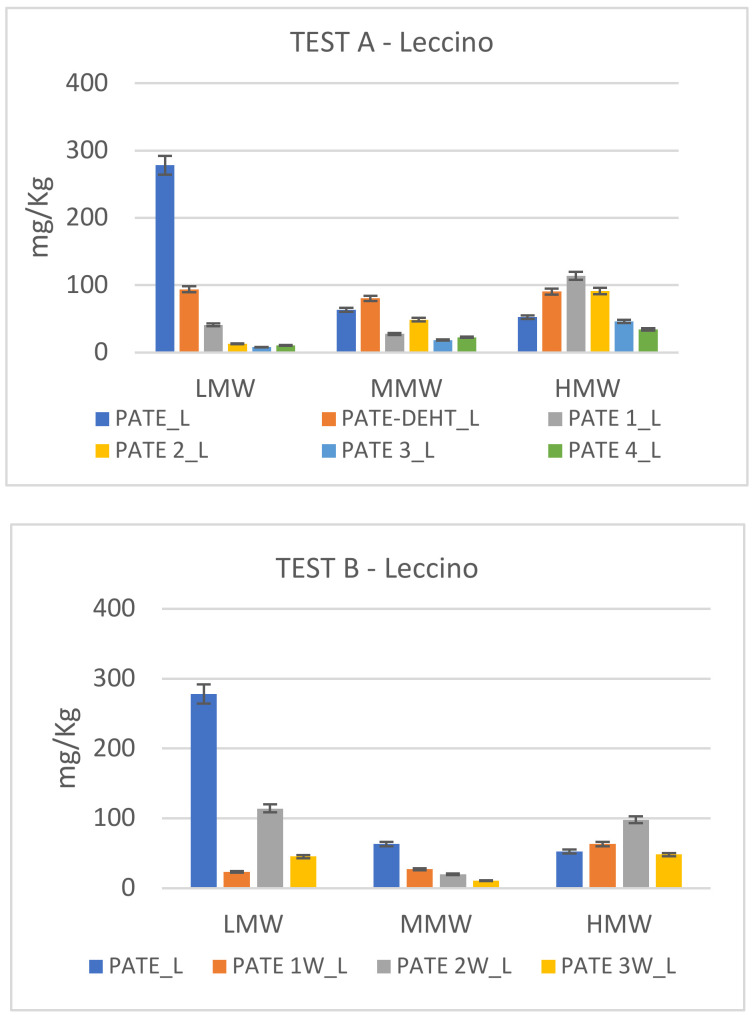
Secoiridoid profile modification in Leccino cv. during chemical debittering (Test A) and microbial fermentation (Test B). Data, expressed in mg/kg, are the mean of duplicate analyses and the coefficient of variation is <5%.

**Figure 3:**
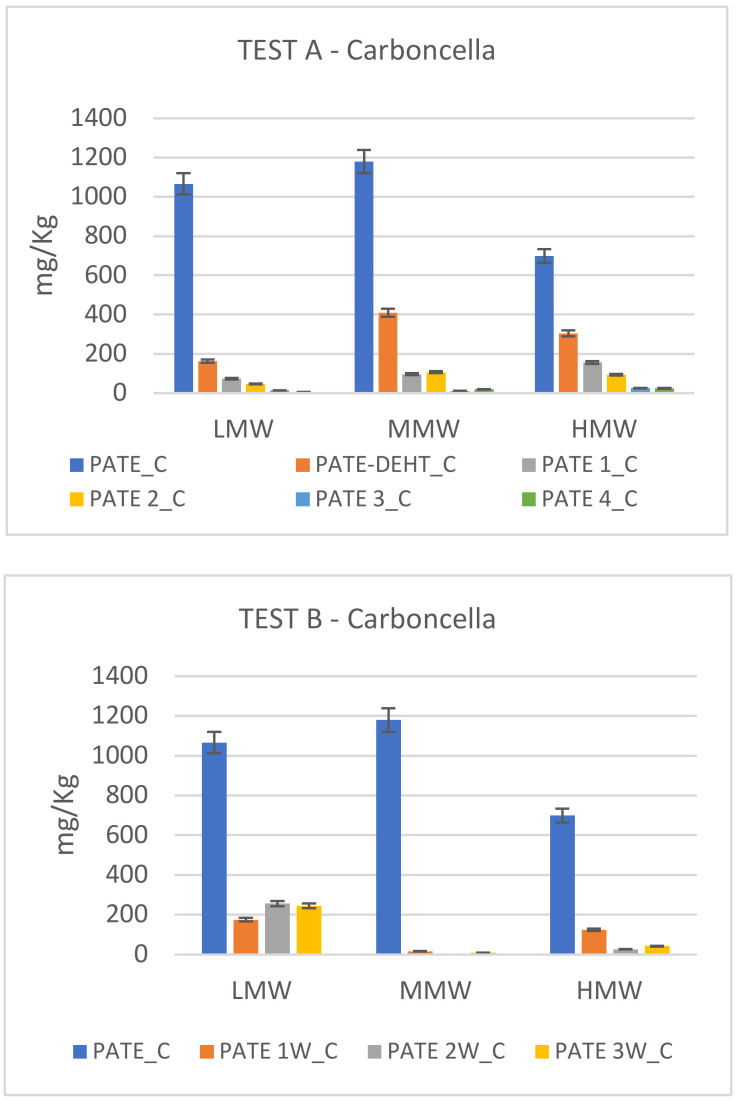
Secoiridoid profile modification in Carboncella cv. during chemical debittering (Test A) and microbial fermentation (Test B). Data, expressed in mg/kg, are the mean of duplicate analyses and the coefficient of variation is <5%.

**Figure 4:**
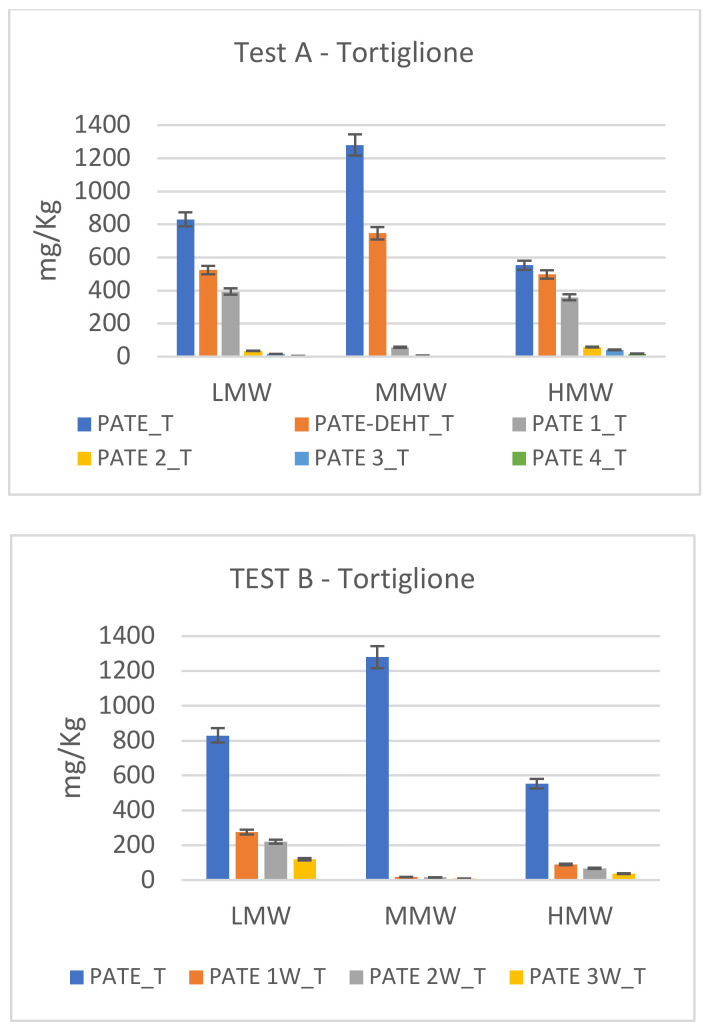
Secoiridoid profile modification in Tortiglione cv. during chemical debittering (Test A) and microbial fermentation (Test B). Data, expressed in mg/kg, are the mean of duplicate analyses and the coefficient of variation is <5%.

In Figure 2, Figure 3 and Figure 4 are showed the different degradation trends during Test A and Test B of phenolics at low (LMW), medium (MMW) and high (HMW) molecular weight. Hydroxytyrosol (HTY), tyrosol (TY) and tyrosyl acetate are phenolics at LMW; 3,4-DHPEA-EDA and p-HPEA-EDA are phenolics at MMW; oleuropein, 3,4-DHPEA-EA, 3,4-DHPEA-EA,H, p-HPEA-EA and p-HPEA-EA,H are HMW.

Figure 2 shows modifications in phenolic pattern of Leccino cv. The secoiridoid profiles differed among each tested filtration. In particular, in both tests, Leccino cultivar shows an initial abundance of low molecular weight phenols which decreases during filtration and microbic degradation. The high molecular weight phenolic compounds instead show an opposite tendency, increasing during the filtrations.

In Figure 3 are showed the different degradation trends during Test A and Test B of secoiridoids at low (LMW), medium (MMW) and high (HMW) molecular weight of Carboncella cv. In the starting pâté, low, medium and high molecular weight phenols are well represented and rapidly drop to values close to zero in both tests. It is possible to notice a drastic decrease already after 24 h in Test A and after one week in Test B. Only LMW in Test B (principally hydroxytyrosol) show a weak rise during the last two weeks.

In Figure 4 are showed the different degradation trend during Test A and Test B of secoiridoids at low (LMW), medium (MMW) and high (HMW) molecular weight of Tortiglione cv. The analyses on the starting pate show a prevalence of MMW phenols in both tests, followed by the low and high molecular weight ones. In Test A, the MMWs already break down after 48 h, while for the LMW and HMW the same situation is reached after 72 h. Test B shows a similar trend, but MMW and HMW show a drastic decrease already after 1 week.

In Table 4 is showed the amount of biophenols that is recovered in the water fractions. The amount of LMW is high principally in Carboncella and Tortiglione cvs. confirming that Leccino cv. is the least rich in bio-phenols although it exhibits the slowest phenolic degradation (Table 3).

### 2.3. Microbial Evolution during Test B

Population dynamics changes of the microbial groups in Test B are illustrated in Table 5. After 21 days, for Leccino cv. was observed a total loss of LAB (from 7.3 × 10⁴ CFU/g to 0 CFU/g). A similar trend of LAB was observed in Carboncella cv. (from 3.0 × 10^4^ CFU/g to 0 CFU/g). The initial LAB count of Tortiglione cv. was much higher (1.8 × 10⁵ CFU/g) than the other cultivars, followed by a significant decline in its population (1.2 × 10⁴ CFU/g). The initial count of Enterobacteriaceae for Leccino, Carboncella and Tortiglione cvs was 4.9 × 10⁴ CFU/g, 1.3 × 10⁴ CFU/g and 2.5 × 10⁴ CFU/g respectively. After 21 days none of these survive. The yeast growth trend for the three cultivars is the following: for Leccino cv. the population was 0 CFU/g at 0 time, reaching a value of 5.6 × 10^3^ CFU/g after 21 days. Regarding Carboncella pâté, there were no different counts that could be detected after 21 days of stirring (from 6.9 × 10⁴ CFU/g to 2.7 × 10⁴ CFU/g). A significant growth was evident in the yeast population of Tortiglione, increasing from 6.1 × 10^3^ CFU/g to 8.5 × 10^5^ CFU/g. Finally, molds showed a constant presence in the two sampling periods for all three cultivars.

### 2.4. Biophenolic Extracts Bioactivity Assays

#### 2.4.1. In Vitro Assays against Multidrug-Resistant Bacteria

None of the pates (Leccino, Carboncella, Tortiglione) showed antibacterial activity at the range of concentrations tested. Indeed, a MIC value of >1024 µg/mL was observed for each extract, regardless of bacterial strain and concentration tested.

The same microorganisms have been tested against some of the most representative molecules present in the extracts (oleuropein and verbascoside and their derivatives, hydroxytyrosol and caffeic acid) and results are shown in Table 6. Oleuropein, hydroxytyrosol and caffeic acid showed borderline activity against *Staphylococcus aureus* Sa1 strain, as indicated by the MIC values of 1024, 256 and 256 µg/mL, respectively. MBC values for oleuropein and HTY overlapped MIC ones thus deposing for a bactericidal anti-staphylococcal effect (Table 6).

#### 2.4.2. Cell Viability and Metabolic Assay

**Figure 5:**
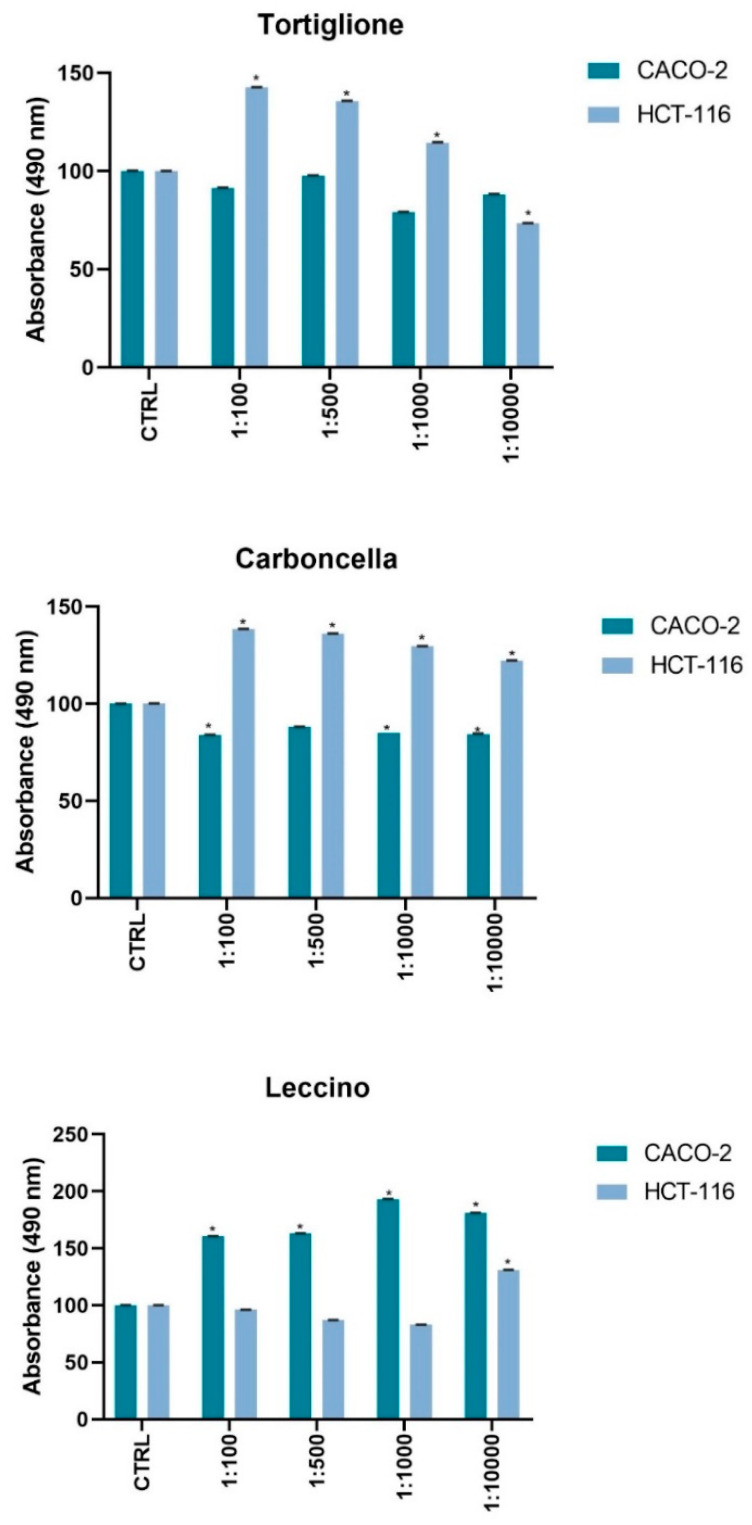
Cell viability and metabolic activity was tested using a colorimetric assay MTS Caco-2 and HCT116 (1.0 × 10^4^ cells/well) were exposed to an increasing dilution (from 1:100; 1:500; 1:1000; 1:10,000) of dried extracts from 1mL *Olea europaea* L. cvs. Leccino, Carboncella and Tortiglione olives at 72 h of times. For each experiment *n* = 5 replicates wells were assayed per clone. Cell viability values were calculated as means and compared to untreated proliferant cells (CTRL). * *p* < 0.001 vs. untreated cells.

We analyzed the bioactivity of the biophenolic extract by MTS assay, on models of human colon cancer cells with different phenotype. The rate of cell proliferation inhibition was peculiar for the three compounds extracted at distinct dilutions (Figure 5). In particular, in Caco2 cells the Leccino extract induces a significant increase (*p* < 0.001) in cell viability at all tested concentrations respect to untreated proliferant cells, a similar effect is observed on HCT116 cells with Carboncella and Tortiglione extract but not for all concentrations. In fact, at maximum dilution (1:10,000) of the Tortiglione extract the vitality of HCT116 seems to be inhibited, while on the Caco2 this treatment did not cause any effect Finally, it is interesting to note that Carboncella extracts induced a reduction in the viability of the Caco-2 cells, *p* < 0.001 (Figure 5).

### 2.5. Gene Expression Assay

**Figure 6:**
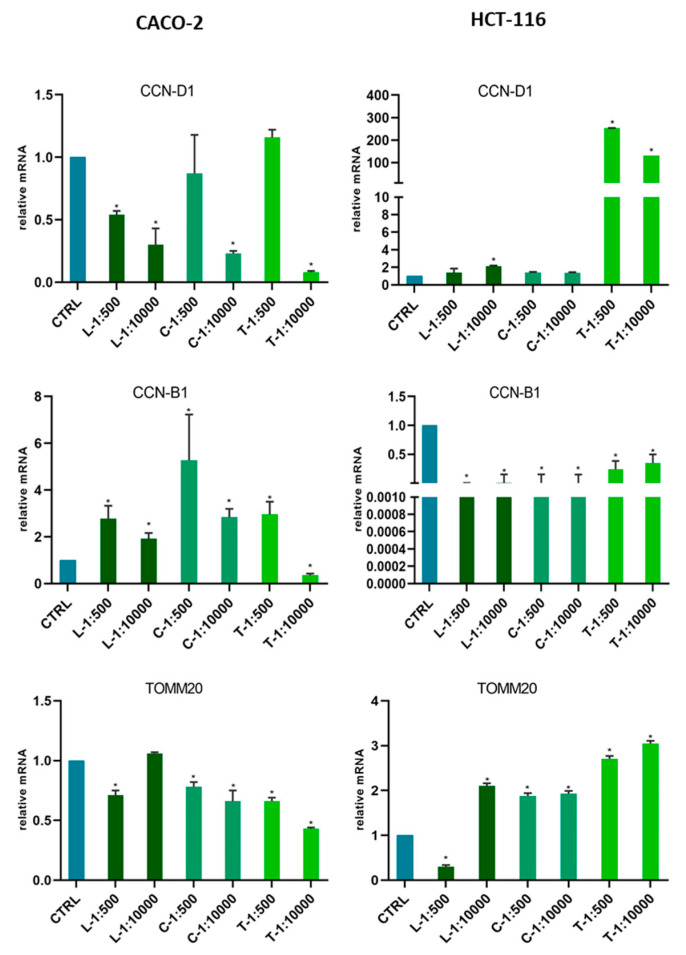
Gene expression modulation under Extract treatments in colon cancer cells Caco-2 and HCT116. Gene expression was analyzed by Real Time-qPCR. The histograms represented normalized data with GUSB gene, and the results showed the average of three independent experiments. CCN-D1 (Cyclin D1); CCN-B1 (Cyclin B1); TOMM20 (Translocase of outer mitochondrial membrane 20). * *p* < 0.05 vs. untreated cells.

In order to evaluate whether the effects on viability variation were related to the modulation of cell cycle phases, or to mitochondrial activity, we tested the gene expression of the characteristic markers of these processes in Caco-2 and HCT116. The lines were treated with 1 mL of Leccino, Carboncella and Tortiglione dried extract for an exposure time of 72 h at different dilutions (1:100; 1:500; 1:1000; 1:10,000). The effects were compared with those of untreated cells (Figure 6).

In colon cell models distinctive, different biological effects were found following the treatments with the three extracts.

In Caco-2 cells, the Tortiglione extract at maximum dilution (1:10,000) induced a significant reduction in mRNA levels for all three genes tested (CCN-D1, CCN-B1 and TOMM20). While in the other model cell, HCT116, the effect of Tortiglione extract induced a strong positive modulation of CCN-D1 and TOMM20. After the treatment with Leccino extracts, the reduction of expression for the CCN-D1 gene was observed in Caco-2. A different behavior was observed in HCT116 cells, indeed a reduction in CCN-B1 expression was induced by all treatments. While, TOMM20 seemed specifically reduced by L-1:500 dilutions in both cell lines. In the case of HCT116, the Tortiglione extract seemed to induce a strong effect on CCN-D1 expression than the other two extracts (Figure 6).

## 3. Discussion

This work showed that spreadable olive by-product (pâté) obtained by DMF technology is a potential source of phenolic compounds. Analyses on the fresh by-product show that the phenolic compounds are not degraded during the oil extraction process, thus remaining in the pâté, they reveal that this by-product is a great source of compounds with possible biological activity [2,3,4]. Lozano-Sanchez et al. [2] found 9270 mg/kg of total phenolic content in pâté obtained by processing an olive batch of “Frantoio” and “Leccino” cvs. together. Cecchi et al. [3] found a total phenolic content >45,000 mg/kg dried pâté for the same cultivars. Tufariello et al. [4] found 7.1 mg/g and 18.1 mg/g for “Leccino” and “Cellina di Nardò” cvs., respectively. We found in two of the cultivars analyzed (Carboncella and Tortiglione) that the amounts of total bio-phenols are very high (>5000 mg/kg; Table 1). Oleacein (3,4-DHPEA-EDA), hydroxytyrosol and verbascoside are the most abundant compounds (Table 2). Similar results are reported by Tufariello et al. [4].

In recent years, it was developed a line of studies on food enrichment [10] with bioactive molecules as bio-phenols obtained from oil processing by-products, as pasta [11] and other bakery products [12], or fish burger enriched with dried olive pomace [13]. The use of this type of waste in food preparation could prove to be a very useful tool for a sustainable development of the olives’ production chain.

Many researches have produced interesting results about the use of by-products as a food ingredient, both for high nutritional value they bring to food and as a valid solution for pollution problems of food processing [14].

On the contrary, to obtain a spreadable pâté, quite like the olive pâté that we find on the market, which is normally obtained from table olives that have been previously de-bittered in brine, pitted and shredded, DMF pâté must be transformed through a process of de-bittering. So, from a by-product as it is, pâté could become a real functional food. Chemical de-bittering of the pâté using diluted sodium solutions (NaOH), after having performed the de-bittering action on the oleuropein, may in this case not be applicable since the soda cannot be easily removed, unlike what happens to the whole fruit processing. To directly obtain a debittered and edible pâté, we compared two methods of phenolic degradation: sequential filtrations and microbial fermentation.

In the Carboncella and Tortiglione pâté the MMW (medium molecular weight secoiridoids) prevail, while for the Leccino pâté the low molecular weight secoiridoids (LMW) prevail.

During Test A (sequential filtrations), Leccino cultivar pâté is the one that loses less phenols and very slowly (Table 2) unlike Carboncella pâté that immediately loses half of the phenols and already at the second filtration (PATE 2) shows a remanence of only 9%.

As regards Test B (microbial fermentation), after the first week, an almost complete degradation of MMW and HMW is noted even if these were present in large quantities in the fresh pâté, while LMW degradation is slower. The pool of endogenous microorganisms was significantly different from the first to the twenty-first day of test B for all three cultivars examined, except for the mold count in all three cultivars and for the yeasts count only in Carboncella cultivar.

The moderate presence of lactic bacteria in the starting patè and the degradation of secoiridoid glycosides assumed that the fermentation that takes place is a lactic fermentation, but the concomitant presence of coliforms and yeasts/molds may have generated also other types of fermentation. We will investigate this aspect in the future with the aim to evaluate the influence of these microorganisms on organoleptic properties of pate during the shelf life.

Sequential filtrations (Test A) represent a faster debittering method compared to fermentation by endogenous and microbial enzymes (Test B).

This work also examines the role of pâté extracts against pathogenic microorganisms and cancer cells.

Phenolic compounds have biological properties and effects (anti-infiammatory, anti-cancer and anti-oxidant activities and inhibition of human LDL oxidation) [15,16], and they could also play an important role in preventing chronic degenerative diseases [17]. Phenolic compounds in the olive fruits, such as oleuropein and hydroxytyrosol, were reported to show antimicrobial activity against viruses, bacteria, yeasts, and fungi [18].

In this study, none of the pâté extracts (Leccino, Carboncella, Tortiglione) showed antibacterial activity against multidrug-resistant Gram-positive and Gram-negative pathogens, regardless of concentrations tested. In this study, we also decided to analyze pure representative compounds present in pâté extracts: oleuropein, hydroxytyrosol, verbascoside and caffeic acid. Despite DMF extracts contain also a good quantity of oleacein, we have decided to evaluate only the antimicrobial effect of its starting homologue (oleuropein) and the simple phenol derived from its degradation (hydroxytyrosol). Instead, the exposure to oleuropein and its hydrolysis derivative (hydroxytyrosol) caused a detectable anti-staphylococcal bactericidal effect. It must be considered that the hypolipemic and antioxidant activity of oleuropein has long been demonstrated [19] and that host fatty acids play a crucial role in the pathogenesis of bacterial infections, such as those caused by *S. aureus*. Indeed, this microorganism possesses metabolic pathways that make it capable to use the fatty acids present in the host’s low-density lipoproteins (LDL) to bypass the chemical and genetic inhibition of bacterial fatty acid synthesis [20]. Further studies aimed at evaluating the effect of *Olea europaea* L. extracts on the invasive capacity of pathogenic bacteria are warranted.

The bioactivity of the biophenolic extracts on cell viability and mitochondrial activity after 72 h from the treatment was peculiar for each of the three extracts, at different dilutions (Figure 5). Indeed, the difference in the observed cell viability ranged from a proliferative effect to a weak cytotoxic effect on different cell lines and for the different varieties of olive pâté. The possible explanation of these results considering the mRNA expression of proteins involved in cell proliferation could have been determined by the differences in the content of the individual phenolic compounds in Leccino, Carboncella and Tortiglione, as shown in Table 2. It was not necessary to consider the stability of these compounds at 37 °C, because the cellular models used mimicked the metabolism of these substances in the human, so as to analyze their full effect after 72 h. D’Antuono et al. [21] in Caco-2 cells showed the presence of derivatives of the compounds that could be attributable to the activity on cellular metabolism of the intestinal epithelium. Carboncella and Tortiglione contained about six times more phenolic compounds than Leccino. In particular, Carboncella and Tortiglione contained significantly higher amounts than Leccino of reported health-promoting phenols concentrations, such as oleuropein (about 40 times), verbascoside (about 20 times), hydroxytyrosol (at least 3 times), and secoiridoid derivative oleacein (more than 40 times) (see Table 2).

In recent years, their biological action from both a food and pharmaceutical point of view has motivated particular attention to the molecular mechanisms underlying their action, although in vitro and in vivo experiments were mostly performed using isolated compounds [22,23]. Among the phenolic compounds present in the complex extracts we tested, oleuropein and hydroxytyrosol were known to improve mitochondrial function to increase energy and biogenesis protecting the cell from excess of oxidative stress [24,25].

Our observations present the first evidence that the complex components of the dry extracts of *Olea europaea* L. cvs. “Leccino”, “Carboncella” and “Tortiglione” can regulate the gene expression of molecules involved in the progression of colorectal tumors. Indeed, Cyclin-D, Cyclin-B1 and TOMM20 play a fundamental role in the progression of the tumor cell cycle. In particular, TOMM20, as a regulator of mitochondrial function, has a direct impact on the production of ATP and contributes to tumorigenic cellular activities including cell cycle regulation. The overexpression of cyclin D1 plays an important role in tumor progression of Colon Rectal Cancer (CRC) and it represents an unfavorable CRC prognostic factor [26]. In the normal intestine and during intestinal tumorigenesis, cyclin D1 expression can be regulated by protein kinase C (PKC) [27]. PKC signal transduction and its intracellular crosstalk influences glucose and lipid metabolism [28]. We have observed an increase in Cycline B1 expression in Caco-2 cells induced by extracts, this may be considered positive, since low Cyclin B1 level is associated with poor prognosis and poor clinical outcome of colorectal cancer [29]. CCNB1 may be candidate diagnostic and prognostic markers, as well as targets for the treatment of CRC. Cyclin B1/CDK1 complex mediate mitochondrial activities in cell cycle progression and stress response as well as metabolism energy reprogramming in tumor adaptive resistance [30]. Translocase of outer mitochondrial membrane 20 (TOMM20) plays an essential role as a receptor for proteins targeted to mitochondria. Overexpression of TOMM20 has been associated with malignant features in colorectal cancer (CRC) cells, while inhibition of its gene expression has led to significant reductions in cell proliferation, migration and invasion [31]. Dry bio-phenolic extracts reduced the expression of tumor progression molecules and mitochondrial activity in the cell models tested. However, their effect has been shown not to depend on their concentration, but rather on their chemical composition. however, these activities will need to be deepened with further studies.

## 4. Materials and Methods 

### 4.1. Olive Pâté Sampling

During October 2019, olives from *Olea europaea* L. “Leccino”, “Carboncella” and “Tortiglione” cvs., were collected in the central area of Italy (Abruzzo Region) and processed by a modern multi-phases centrifugal plant (Leopard DMF 4 Series, Pieralisi Group S.p.A. Jesi, Italy) located in the Oleificio Di Berardino (Nereto, TE, Italy). The very-wet pomace (pâté) samples were placed in thermal bags and quickly brought to the laboratory, where they were immediately frozen and then stored at −20 °C until use.

### 4.2. Characterization of Fresh DMF Pâté

The pH of pâté was measured with an Istek pH Meter 730P model (Istek, Inc., Seoul, Korea).

The titratable acidity was determined by titrating 20 g of pâté with NaOH 0.1 N solution using phenolphthalein as an indicator 1% (*w*/*v*) solution in ethanol. Results are expressed in grams of citric acid per 100 g of fresh pâté.

The moisture content was determined by a drying process. Homogenized samples (about 20 g) have been placed and weighed in a Pyrex crucible, previously dried at 105 °C up to a constant weight. The samples have been put in an oven at 105 °C for at least 24 h and until the weight was constant. The moisture content was calculated as the difference in weight.

To determine ash content, homogenized samples (about 5 g) have been placed and weighed in a porcelain crucible, previously dried at 105 °C up to a constant weight. Then, they have been heated to 550 °C in a thermostatically controlled muffle furnace for at least 24 h and until reaching a whitish brown color.

The oil content was determined by Soxhlet extractor. The oil was extracted from the dried olive pulp used for moisture determination by repeated washing (percolation) with petroleum ether 40–60, under reflux in a special glassware. The ether was removed by evaporation and the residual oil was weighed.

### 4.3. Test A (Pâté de-Bittering through Daily Filtering)

**Figure 7:**
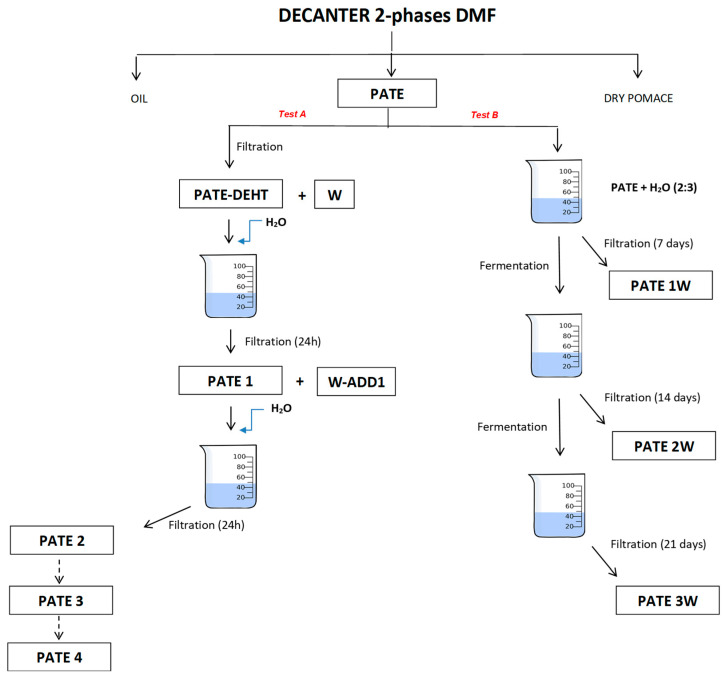
Experimental trial.

Test A started with the filtration of pâté samples (PATE_L: pâté from Leccino cv.; PATE_C: pâté from Carboncella cv.; PATE_T: pâté from Tortiglione cv.) to obtain PATE_DEHT (L, C and T for Leccino, Carboncella and Tortiglione, respectively) and water (W). The filtered product was mixed with an equal amount of water (W-ADD1) and, after 24 h, was filtered. From this second filtration it was obtained PATE 1 (L, C and T for Leccino, Carboncella and Tortiglione, respectively). After 24 h the same procedure was repeated, maintaining the same proportion between pâté and water, until fifth filtration (PATE 2, PATE 3, PATE 4; L, C and T for Leccino, Carboncella and Tortiglione, respectively). The experiment was stopped after five days (Figure 7). For each step of filtration, it was used a vacuum pump coupled with an apparatus for membrane filtration equipped with a filter consisting of an 80-denier nylon mesh pantyhose. Pâté from each step of filtration was used for determination of the phenol content. We conducted tests at room temperature to recreate the real conditions of an oil mill as much as possible.

### 4.4. Test B (Pâté de-Bittering by Indigenous Microflora)

Test B started with 300 mL of pâté mixed in a beaker with 200 mL of water (3:2) and placed on the magnetic stirrer mixer. After 1 week of natural fermentation, 100 mL of this solution were taken and filtered as above (PATE 1W; L, C and T for Leccino, Carboncella and Tortiglione, respectively). Same procedure was repeated after 2 and 3 weeks (PATE 2W and PATE 3W; L, C and T for Leccino, Carboncella and Tortiglione, respectively) (Figure 7). The test was carried out at room temperature, respecting the conditions of maximum sterility to preserve the endogenous microbial pool of the pâté. To monitor the microbial biodiversity at the start and at the end of experiment, serial dilutions of samples at time 0 and after 21 days were plated on agar media: Enterobacteriaceae were grown on MacConkey Agar (Oxoid, Basingstoke, UK) incubating the plates at 37 °C for 48 h; yeasts and molds on Malt Extract Agar (MEA; Oxoid) at 30 °C for 48 h and 5 days, respectively; lactic acid bacteria on Man, De Rogosa and Sharpe (MRS; Oxoid) at 30 °C for 48 h in anaerobic atmosphere. Culture responses were expressed as colonies forming units (CFU) per gram of pâté. Pâté from each weekly filtration was used for determination of the phenol content.

### 4.5. Preparation of Pâté Extracts and Characterization of the Biophenolic Pattern

The pâté extracts were prepared according to the International Olive Council method [32], adapted as explained below. 1 g of homogenized olive pâté was transferred in a test tube with 1 mL of internal standard (syringic acid 15 mg/L) and vortexed for 30 s. The mixture was extracted with 5 mL of methanol/water (80/20), vortexed for 1 min, sonicated in an ultrasonic bath for 15 min and centrifuged at 3500 rpm for 5 min. An aliquot of methanolic/water solution was filtered in PVDF 0.45 µm. The HPLC analysis of the phenolic extracts was carried using a LC 200 high resolution liquid chromatograph, equipped with a Series 200 UV/Vis detector (Perkin Elmer, Waltham, MA, USA), a 7725 Rheodyne injector, a 20 µL sample loop and a Totalchrom workstation (Perkin Elmer, Waltham, MA, USA) for data acquisition. Separation on Spherisorb ODS2 (250 × 4.6 mm I.D., 5 µm; Waters, Milford, MA, USA) was performed at 25 °C under a constant flow rate of 1 mL/min and the following ternary gradient program (in %): t = 0 min, A (water 0.2% H_3_PO_4_ (V/V))/B (methanol)/C (acetonitrile) = 96/2/2 *v*/*v*/*v*; t = 40 min, A/B/C = 50/25/25 *v*/*v*/*v*; t = 45 min, A/B/C = 40/30/30 *v*/*v*/*v*; t = 60 min, A/B/C = 0/50/50 *v*/*v*/*v*; t = 70 min, A/B/C = 0/50/50 *v*/*v*/*v*; t = 72 min, A/B/C = 96/2/2 *v*/*v*/*v*; t = 82 min, A/B/C = 96/2/2 *v*/*v*/*v*. Eluted compounds were detected at 280 nm. First, 20 µL of the external calibration standard solution were injected to calculate the values of the response factors for 1 µg of tyrosol and 1 µg of syringic acid. Finally, 20 µL of the sample solution were injected into the HPLC system. Compounds identification was made using syringic acid as internal standard.

### 4.6. Biophenolic Extract Bioactivity Assays

The extracts were resuspended in distilled water and sterilized by filtration with 0.22 µm STARLAB’s Syringe Filters PVDF (sterile).

#### 4.6.1. In Vitro Antimicrobial Assays against Multidrug-Resistant Bacteria

The antibacterial activity of each extract and of the most representative molecules present in each extract (oleuropein, verbascoside, hydroxytyrosol and caffeic acid) was evaluated in vitro against bacterial strains representative both for Gram-positive and Gram-negative pathogens: *Pseudomonas aeruginosa* AC12a, *Staphylococcus aureus* Sa1, *Enterococcus faecalis* ATCC 29,212 and *Escherichia coli* K2P. Oleuropein and caffeic acid were purchased at Sigma-Aldrich (St. Louis, MO, USA), verbascoside at HWI Pharma Service GmbH (Rülzheim, Germany) and hydroxytyrosol at Extrasynthese (Genay, France).

The minimum inhibitory concentration (MIC) and minimum bactericidal concentration (MBC) of each extract were determined by the broth microdilution method according to the Clinical Laboratory Standards Institute guidelines [33], with some modifications. Briefly, serial twofold dilutions (1024–2 µg/mL) of each extract were prepared in Cation-adjusted Muller-Hinton broth (CAMHB) (Oxoid srl; Garbagnate M.se, Milano, Italy) in 96-well microtiter plates (ANICRIN srl; Scorzè, Venezia, Italy), while control wells contained CAMHB only. Each well, containing a final volume of 100 µL, was then inoculated with 5 µL of a standardized inoculum prepared to test each extract at three different bacterial concentrations (0.5–1 × 10^3^, 0.5–1 × 10^4^ and 0.5–1 × 10^5^ CFU/well). MIC was read as the lowest concentration of the extract that completely inhibited visible growth following 24 h-incubation at 37 °C under aerobic atmosphere. MBC was also assessed by plating 100 µL of broth from clear wells of MIC microtiters onto Muller-Hinton agar plates. Following 20 h-incubation at 37 °C under aerobic atmosphere, MBC was defined as the lowest concentration of the test agent killing at least 99.99% of the original inoculum.

#### 4.6.2. Test on Colon Cancer Cells

##### Human Colon Cell Culture and Treatments

For this study we used two colon cancer cell lines with different degrees of differentiation, Caco-2 as a model for the differentiated phenotype and HCT116 for the cell type with a low degree of differentiation [34]. The lines were obtained from American Type Culture Collection (ATCC) (Manassas, VA, USA). HCT116 and Caco-2, were cultured at 37 °C in DMEM medium containing 10% and 20% Fetal Bovine Serum (FBS) respectively, 100 U/mL penicillin/streptomycin and 2 mM L-glutamine (EuroClone, Pero, MI, Italy). For the experiments, in cells grown at approximately 75% confluence, the cells were treated with dried extracts from 1 mL *Olea europaea* L. cvs. Leccino, Carboncella and Tortiglione olives, for 72 hours’ exposure time at different dilutions (1:100; 1:500; 1:1000; 1:10,000).

##### Cell Viability and Metabolic Assay

For cell viability and metabolic activity was tested using a colorimetric assay, HCT116, SW480 and Caco-2 cells were cultured in 96-well plate at a concentration of 1.0 × 104 cells/well. Cells were exposed to an increasing dilution (from 1:100; 1:500; 1:1000; 1:10,000) of dried extracts from 1mL *Olea europaea* L. cvs. Leccino, Carboncella and Tortiglione olives at 72 h of times. This was followed by incubation with 10 µL/well of 2-[2-methoxy-4-nitrophenyl]-3-[4-nitrophenyl]-5-[2,4-disulphophenyl]-2H-tetrazolium, monosodium salt (MTS) assay (Promega, Madison WI, USA) a 37 °C for 1 h, the absorbance was measured at 490 nm with a microplate reader by GloMax-Multi Detection System (Promega). For each experimental condition, seven repetitions were performed in three independent experiments.

##### Real-Time Quantitative PCR Analysis (qRT-PCR)

Total RNA was isolated from Caco-2 and HCT116 cells treated as specified in “Cell Culture and treatments”, using TriFast (EUROGOLD EuroClone) according to the manufacturer’s instructions. RNA samples were assessed for purity and quantified by Nanodrop 1000 Spectrophotometer (Thermo Fisher Scientific). The synthesis of complementary DNA (cDNA) was performed employing the GoTaq^®^ 2 Step RT-qPCR Kit (Promega) according to the manufacturer’s instructions. The mRNA levels were evaluated by SYBR Green quantitative real-time PCR (qRT-PCR) analysis using StepOne™ 2.0 (Applied Biosystems, Thermo Fisher Scientific, Waltham, MA, USA). Data were analyzed using the comparative Ct method and were graphically indicated as 2−ΔΔCt + SD. In accordance with the method, the mRNA amounts of the target genes were normalized by the ratio on the median value of the endogenous housekeeping gene (GUSB) obtained in each treated cells vs. untreated (proliferant) cells.

Targets and reference genes were amplified in triplicate in a volume of 10 μL containing 1 μL template cDNA, 0.2 μL of primers mixture and 5 μL of GoTaq^®^ 2-Step RT-qPCR System (Promega) according to the manufacturer’s instructions. The cycling conditions were performed as follows, 10 min at 95 °C and 40 cycles of 15 s at 95 °C followed by 1 min at 60 °C and final elongation of 15 s at 95 °C. Gene expression levels were analyzed for the Translocase of Outer Mitochondrial Membrane 20 (TOMM20) and for the cell cycle markers: cyclin D1 (CCND1) whose activity is required for cell cycle G1/S transition and cyclin B1(CCNB1) whose levels are maximal at G2/M. The sequences of paired oligonucleotides were: 5’-GACCCCAACTTCAAGAACAG-3’ and 5’-TGGTCTACGCCCTTCTCATA-3’ for TOMM20; 5’-AGGAGCTGCTGGTAACCACT-3’ and 5’-TGTGTTCGCAGCAAATGGAG-3’ for CCND1; 5’-GGAGGGGACTCACCAAGAGA-3’ and 5’-CGATGTGGCATACTTGTTCTTG-3’ for CCNB1.

### 4.7. Statistical Analyses

The results were subjected to T-test analysis; statistical significance was set at *p* < 0.05 and *p* < 0.001.

## 5. Conclusions

The present work investigated the efficacy of two different ways of debittering pâté from DMF decanter and the effects of bio-phenolic extracts on pathogenic microorganisms and cancer cells.

Daily filtrations of pâté of the three cultivars have been shown to be more efficient in phenolic degradation. The activity of the indigenous microflora, on the other hand, takes a longer time to degrade the phenolic component and therefore to de-bitter it. The results reported indicate a possible enhancement of this debittered by-product, especially as an ingredient in food preparation. Large-scale experiment is planned to improve and validate the use of these debittering techniques.

In this study, none of the pâté extracts showed antibacterial activity on multidrug-resistant bacteria in the tested concentration range.

Bio-phenolic dry extracts reduced the expression of molecules that are decisive in tumor progression and mitochondrial activity. However, their effect has showed to be not dependent on their concentration, but rather on the relative chemical composition.

Further studies are needed to identify molecular pathways of tumor suppressive effect by extracts of tree products of *Olea europaea* L. to explore the possibility of its therapeutic potential.

## Figures and Tables

**Table 1:** Chemical characteristics of DMF fresh pâté. Significant differences are indicated by different letters at *p* < 0.05 (*t* test).

	Leccino	Carboncella	Tortiglione
Titratable acidity ^1^	0.29 a	0.47 b	0.56 c
pH	5.28 a	5.13 b	4.96 c
Moisture ^2^	84.10 a	81.60 b	86.01 c
Ash ^3^	1.57 a	2.75 b	2.18 c
Oil content ^3^	4.00 a	4.20 b	3.70 c
Total biophenols ^4^	948 a	5899 b	5543 c

^1^ g/100 g of pâtè, as citric acid; ^2^ g of H_2_O/100 g of pâè; ^3^ g/100 g of pâtè; ^4^ The total content of biophenols includes the unidentified eluted compounds without oxidized forms. It is expressed as mg/kg of pâté.

**Table 2:** Amount of individual identified phenolic compounds in DMF fresh pâté. Data are expressed in mg/kg of pâté. Significant differences are indicated by different letters at *p* < 0.05 (*t* test).

Phenolic Compound	Leccino	Carboncella	Tortiglione
3,4-DHPEA (Hydroxytyrosol)	243 a	977 b	720 c
*p*-HPEA (Tyrosol)	21 a	57 b	67 c
*p*-Hydroxybenzoic acid	30 a	197 b	188 b
Vanillic acid	29 a	87 b	93 b
Caffeic acid	0 a	15 b	97 c
Vanillin	22 a	40 b	20 a
*p*-Cumaric acid	5 a	149 b	13 c
Hydroxytyrosyl acetate	5 a	36 b	122 c
Ferulic acid	43 a	61 b	30 c
Verbascoside	42 a	788 b	995 c
*o*-Cumaric acid	1 a	12 b	59 c
3,4-DHPEA-EDA (Oleacein)	24 a	1050 b	1249 c
Oleuropein	10 a	391 b	472 c
3,4-DHPEA-EA (Oleuropein aglycone)	4 a	24 b	50 c
Tyrosyl acetate	15 a	32 b	43 c
Rutin	16 a	374 b	112 c
*p*-HPEA-EDA (Oleocanthal)	39 a	130 b	31 a
Pinoresinol, 1-Acetoxypinoresinol	9 a	146 b	10 a
Cinnamic acid	2 a	8 b	73 c
*p*-HPEA-EA (Ligstroside aglycone)	26 a	203 b	7 c
Luteolin	0 a	61 b	88 c
3,4-DHPEA-EA,H	6 a	68 b	23 c
Apigenin	0 a	6 b	13 c
Methyl-luteolin	0 a	0 a	5 b
*p*-HPEA-EA,H	6 a	13 b	2 c

**Table 3:** Comparison of degradation rate of total phenolic compounds during test A and test B. Data are expressed as total biophenol content mg/kg of DMF pâté and, between parenthesis, the remaining percentage of total biophenols.

Test A				Test B			
Steps	Leccino	Carboncella	Tortiglione	Steps	Leccino	Carboncella	Tortiglione
PATE	948 (100%)	5899 (100%)	5543 (100%)	PATE	948 (100%)	5899 (100%)	5543 (100%)
PATE-DEHT	815 (86%)	2528 (43%)	4051 (73%)	PATE 1W	447 (45%)	1346 (23%)	3180 (57%)
PATE 1	662 (70%)	1021 (17%)	1110 (20%)	PATE 2W	446 (45%)	664 (11%)	1230 (22%)
PATE 2	553 (58%)	536 (9%)	252 (5%)	PATE 3W	417 (45%)	645 (11%)	580 (10%)
PATE 3	214 (23%)	140 (2%)	126 (2%)				
PATE 4	183 (19%)	140 (2%)	93 (2%)				

**Table 4:** Recovery of biophenols in water fractions. Data, expressed in mg/L, are mean ± standard deviation.

	W_L	W_C	W_T	W-ADD1_L	W-ADD1_C	W-ADD1_T
LMW	134 ± 7	389 ± 19	579 ± 29	55 ± 3	302 ± 15	241 ± 12
MMW	21 ± 1	197 ± 10	145 ± 7	0.0 ± 0.0	167 ± 8	6.4 ± 0.3
HMW	26 ± 1	73 ± 4	28 ± 1	4.7 ± 0.3	104 ± 5	30 ± 2

**Table 5:** Microbial monitoring. Data are expressed in CFU/g. Significant differences are indicated by different letters at *p* < 0.05 (*t* test). 0 d = 0 days; 21 d = after 21 days.

Microorganisms	Leccino		Carboncella		Tortiglione	
	0 d	21 d	0 d	21 d	0 d	21 d
Lactic acid bacteria	7.3 × 10^4 ^ a	0 b	3.0 × 10^4^ a	0 b	1.8 × 10^5^ a	1.2 × 10^4^ b
Enterobacteria	4.9 × 10^4^ a	0 b	1.3 × 10^4^ a	0 b	2.5 × 10^4^ a	0 b
Yeasts	0 a	5.6 × 10^3^ b	6.9 × 10^4^ a	2.7 × 10^4^ a	6.1 × 10^3^ a	8.5 ×10^5^ b
Moulds	2.6 × 10^3^ a	1.2 × 10^3^ a	1.2 × 10^3^ a	1.8 × 10^3^ a	1.1 × 10^4^ a	3.2 × 10^4^ a

**Table 6:** In vitro activity of oleuropein, verbascoside and derivatives (hydroxytyrosol and caffeic acid, respectively) against bacterial strains representative for Gram-positive and Gram-negative pathogens. The minimum inhibitory concentration (MIC) and minimum bactericidal concentration (MBC) values of each compound were determined by the broth microdilution method and expressed as µg/mL.

Bacterial Strain	Oleuropein		Verbascoside		Hydroxytyrosol		Caffeic Acid	
	MIC	MBC	MIC	MBC	MIC	MBC	MIC	MBC
*P. aeruginosa AC12a*	>1024	>1024	>1024	>1024	>256	>256	>256	>256
*S. aureus Sa1*	**1024**	**1024**	>1024	>1024	**256**	**256**	**256**	>256
*E. faecalis ATCC29212*	>1024	>1024	>1024	>1024	>256	>256	>256	>256
*E. coli K2P*	>1024	>1024	>1024	>1024	>256	>256	>256	>256
